# Characterizing pollution and source identification of heavy metals in soils using geochemical baseline and PMF approach

**DOI:** 10.1038/s41598-020-63604-5

**Published:** 2020-04-15

**Authors:** Hui-Hao Jiang, Li-Mei Cai, Han-Hui Wen, Jie Luo

**Affiliations:** 1 Key Laboratory of Exploration Technologies for Oil and Gas Resources (Yangtze University), Ministry of Education, Wuhan, 430100 China; 2 College of Resources and Environment, Yangtze University, Wuhan, 430100 China; 3 Key Laboratory of Mineralogy and Metallogeny, Guangzhou Institute of Geochemistry, Chinese Academy of Sciences, Guangzhou, 510640 China; 4 No.940 Branch of Geology Bureau for Nonferrous Metals of Guangdong Province, Qingyuan, 511500 China

**Keywords:** Environmental sciences, Environmental chemistry

## Abstract

It is necessary to establish local geochemical baseline concentrations (GBCs) due to the lack or the inapplicability of regional background values in the study area. The establishment of GBCs of heavy metal (HM) in soil helps in making the accurate assessment of pollution, and then provides a basis for pollution control. Based on this, a case study was undertaken to study the GBCs of the Jiedong District, Guangdong Province, China. In this research, cumulative frequency distribution curves were utilized to determine the local GBCs in the subsoils. The determined GBCs of Cr, Hg, As, Pb, Ni, Cd, Cu, Zn, Co and V were 39.91, 0.072, 11.48, 47.62, 12.70, 0.17, 14.22, 64.54, 6.31, and 68.14 mg/kg, respectively. The average concentrations of Hg, As, Pb, Cd, Cu and Zn in the topsoils exceeded the corresponding baseline concentrations. In particular, the contents of Cd and Hg were 1.53 and 2.22 times higher than GBCs. According to this baseline criterion, enrichment factor (EF), pollution load index (PLI) and ecological risk index (RI) were applied to assessing HM pollution. EF and PLI suggested that most areas were under moderate contamination, while Hg and Cd pollution was more serious. And the RI values presented that the potential ecological risks were low in most parts of the study area. The possible origins of HMs were identified by combining positive matrix factorization with EF and geostatistics. Comprehensive analysis indicated that Hg and Cd were related to industrial activities, such as textile and garment processing, plastic and rubber production and metal manufacturing. Arsenic and part of Cu mainly came from agricultural activities, namely the use of pesticides, fertilizers and livestock manures. Lead and Zn were mainly attributed to traffic emissions. Chromium, Ni, V, Co, and part of Cu were originated from natural source controlled by parent materials. The corresponding contributions of these sources were 20.61%, 24.20%, 19.22% and 35.97%, respectively. This work provides information to prevent and control the soil HM pollution by proposing the efficient management of anthropogenic sources.

## Introduction

In recent years, soil pollution has become increasingly severe due to rapid industrialization and the increase of urban population^1–3^. Soil heavy metal (HM) pollution has received wide concern owing to the strong toxicity, bioavailability and persistence of HMs^4^. The high content of HMs in soil not only directly affects nutrients, structure and function of soil, reduces the biological activity of soil, but also adversely affects food quality and human health^5,6^. HMs in the soil pose a grave threat to human body through skin contact, inhalation and ingestion^7^. For example, cadmium can cause cardiovascular and cerebrovascular diseases and renal dysfunction^8^. Long-term exposure to lead-contaminated environments can result in serious damage to nerves, hematopoietic systems, and kidneys^9^. Therefore, HM pollution in soil is particularly concerned worldwide and urgently needs to be resolved.

Determining the pollution level and potential sources is the key to solving the problem of HM contamination in soil^11,11^. Geochemical baseline concentrations (GBCs) refer to the natural levels of HMs in the soil that do not include the effects of anthropogenic activities^12,13^. On the one hand, the background values (BVs) generally are reference values for a wide range of areas, which may not be applicable for specific small-scale study areas. On the other hand, many areas lack BVs. Therefore, it is necessary to establish local GBCs^14,15^. The result of some widely used quantitative indicators in pollution assessment, such as ecological risk index (RI), pollution load index (PLI), contamination factor (CF), and enrichment factor (EF), will be more accurate when regional GBCs are used. As a result, establishing GBCs on a local scale is a priority to better assess HM contamination^16^. The two major origins of soil HMs are natural and anthropic sources^17^. The natural origin of HMs is related to soil parent materials, and anthropic sources are associated with various anthropogenic inputs, covering industrial production, agricultural activities and traffic emission^18^. Quantitative apportionment of possible origins of soil HMs is extremely important for the prevention and supervision of pollution^19^. Quantitative methods for source apportionment mainly included receptor models like absolute principal component scoring, chemical mass balance method and positive matrix factorization model (PMF). PMF method has been extensively applied to origin apportionment of atmosphere, sediment and water^20–22^. Recently, some achievements have been conducted in identifying the source of soil contaminants^1,17,23^. Apportioning HM sources in soils by PMF model is of great value to understand the characteristics and contributions of different origins. Hence, local authorities can take valid measures to decrease pollution emissions.

Over the years, the Hanjiang Delta (including Jiedong District), the second biggest economic zone after the Pearl River Delta in Guangdong Province, has witnessed rapid economic development. With this prerequisite, Jiedong District experienced fast development and industry optimization, forming an industrial structure with textile and garment, chemical plastics and metal manufacturing as the pillars. As to agriculture, cultivation and production have boomed. To be more specific, rice and other food crops have been planted as well as a large number of fruits such as litchi, longan and banana. In addition, its traffic advantages are also obvious. There are two expressways and two national highways running through the whole district. Those factors above: industrial activities, agricultural practices and heavy traffic in the study area provided a variety of sources of HM emissions. There are many studies on the evaluation and origin identification of soil HM contamination in counties and districts^5,24,25^, but few on the establishment of local GBCs for contamination evaluation.

The major targets of this study were: (1) to determine the GBCs of HMs in soil of the study area; (2) to evaluate HM pollution levels in combination with EF and PLI, and assess potential ecological risks by RI; (3) to quantitatively identify the possible sources of HMs using PMF model. This research provides a reference for establishing GBCs to more accurately assess HM pollution and source apportionment in those areas where local background values are lacking or inapplicable.

## Materials and methods

### Study region

**Figure 1:**
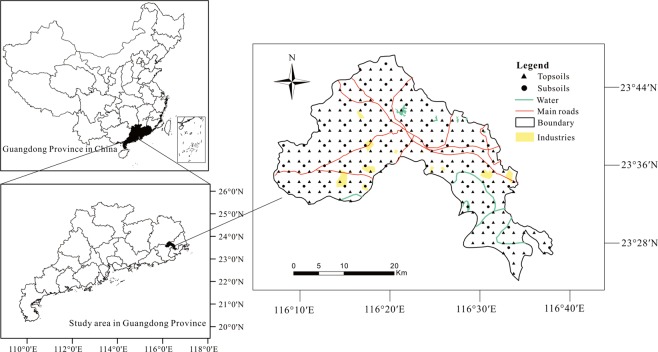
Location of the Jiedong District and distribution of sampling sites.

Jiedong District is situated at the east part of Guangdong Province, South China (Fig. 1), which covers approximate 680 km^2^ and has a population of 1.2 million. The area has a subtropical monsoon climate, with annual mean precipitation and temperature of 1722 mm and 21.5 °C, respectively. Relying on the production base, Jiedong carries out the planting and processing of agricultural and sideline products. The main economic crops in the region cover bananas, citrus, longan and bamboo shoots. The industrial development of Jiedong District has formed an industrial layout of “one district and six parks” with the economic development zone as the leader and the six township industrial parks under the backbone.

### Sample collections and analysis

In this research, we collected 212 topsoil (0–20 cm) and 52 subsoil (150–200 cm) samples from Jiedong District (Fig. 1). The sampling densities of topsoil and subsoil were 4 km^2^ and 16 km^2^, respectively. Each soil sample was a synthesis of several sub-samples within 100 meters of the nearby sample site. All samples were gathered in polyethylene bags and brought back for chemical analysis. These samples were dried under daylight, and passed via 2.0 mm nylon sifters^26^. Contents of 8 HMs (Cr, Pb, Ni, Cu, Zn, Co, V and Ti) were measured by XRF^18^. Soil samples were treated with KMnO_4_-H_2_C_2_O_4_ and the contents of As and Hg were determined by AFS^17^. Besides, partial samples were treated using HNO_3_-HF-HClO_4_, and the content of Cd was determined by ICP-MS. We used standard references materials (GSS-1), replicate samples and reagent blanks for quality control. Four standard references, two blank samples and four duplicate samples were added to each batch of 50 samples. The relative standard deviations of repetitive sample did not exceed 5%. The method detection limits (MDL) for Cr, Pb, Ni, Cu, Zn, Co, V, Ti, As, Hg and Cd were 2, 1, 1, 1, 2, 0.8, 3, 10, 0.05, 0.0003 and 0.02 mg/kg, respectively.

### Geochemical baseline concentrations

Geochemical baseline offers a method of identifying the natural and anthropic sources of HMs in soils and also play a critical role in assessment of HM pollution^13,27^. Subsoil samples (150–200 cm) were utilized to estimate the baseline concentrations of HMs in this study because they are largely unaffected by human activities. Furthermore, baseline concentrations were determined by applying the cumulative frequency distribution (CFD) curves^15^. Kolmogorov–Smirnov (K-S) test was performed to verify the content distribution of each HM before plotting the CFD curves^26,28^. For CFD curves, the x axis shows the cumulative frequency, and the y axis shows the log-transformed values of the HM content or HM contents. And then the linear part of the CFD curve between the two inflection points is then applied to identify the baseline content of each HM. In this research, the inflexions in a curve were defined under the linear regression criteria for P < 0.05 and R^2^ > 0.95, and the extreme values were removed until the rest of data met the criteria of linearity^15^. The baseline values were determined by the following rules: If the curve had an inflection point, the baseline value was gained from the data below the inflection point; if the curve had two inflection points, the similarity of the data distribution between the two inflection points defined which inflection point was used as the basis for calculation. Finally, the data below the selected inflection point was averaged to obtain the baseline values.

### Evaluation of soil contamination

To assess the contamination of HM in soil, EF and CF of selected HM as well as PLI of each sample site were calculated. EF is applied to evaluate the accumulation level of soil HMs. It is computed by Eq. (1) ^29^:

1 $${\rm{EF}}=\frac{{({{\rm{C}}}_{i}/{{\rm{B}}}_{ref})}_{topsoils}}{{({{\rm{C}}}_{i}/{{\rm{B}}}_{ref})}_{GBCs}}$$

where C_i_ represents the content of HM, and B_ref_ represents the content of the reference metal. Generally, elements Al, Mn, Fe, Ti and Zr are chosen as references^30^. In the study, Ti was selected due to the less impact of human activities^18^. Based on the EF value, the enrichment classification of HM is as follows: negligible accumulation (EF < 1); minor accumulation (1~2); moderate accumulation (2~5); major accumulation (5~20); high level accumulation (20~40); and exceedingly high accumulation (EF ≥ 40)^23^.

CF and PLI were applied to evaluate the HM contamination in soils. CF_i_ is the rate of the content of HM i to its corresponding GBC, and it shows the contamination level with a single HM, as given below^31^:

2 $$C{F}_{i}=\frac{{C}_{i}}{{C}_{GB}}$$

CF_i_ is used to calculate the PLI value, which is computed via the following expression:

3 $$PLI=\sqrt[n]{C{F}_{1}\times C{F}_{2}\times C{F}_{3}\times \cdot \cdot \cdot \cdot \cdot \cdot \times C{F}_{n}}$$

Here, C_i_ represents the contents of HM i and C_GB_ represents the GBC of HM i. According to the research of Tomlinson *et al*.^32^, PLI > 1 demonstrates HM contamination. Furthermore, the pollution levels are delimited as follow: no contamination (PLI < 1); moderate contamination (1~2); severe contamination (2~5); extreme contamination (PLI ≥ 5).

The RI presented by Hakanson^33^ is performed to investigate ecological risks caused by HMs. The RI is computed using below expression:

4 $$RI=\mathop{\sum }\limits_{i=1}^{n}{E}_{i}$$

5 $${E}_{i}={T}_{i}\,\ast \,C{F}_{i}$$

here, E_i_ represents a single risk factor of HM i, and T_i_ represents the toxic-response factor of HM i. The values of T_i_ for Zn, V, Cr, Co, Pb, Ni, Cu, As, Cd and Hg were 1, 2, 2, 5, 5, 5, 5, 10, 30 and 40, respectively. Where CF_i_ is the contamination factor of the HM i, which mentioned in Eq. (2). The RI could be calculated as the total of the E_i_. Ecological risks based on the RI are divided into four categories^33^: RI < 150 suggests a low ecological risk; 150~300 suggests a medium ecological risk, 300~600 suggests a significant ecological risk; and RI > 600 suggests an extreme ecological risk.

### PMF model

PMF was proposed and developed by Paatero and Tapper^34^, which is a multivariate receptor model for source apportionment. Based on EPA PMF 5.0 user manual:

6 $${{\rm{x}}}_{ij}=\mathop{\sum }\limits_{k=1}^{p}{G}_{ik}{F}_{kj}+{E}_{ij}$$

here, x_ij_ is a data matrix of HM j in sample i; G_ik_ is the contribution matrix of source k for sample i; and F_kj_ is a factor profile of HM j for source factor k. E_ij_ represents the residual for HM j from sample i, and it can be acquired through minimizing the target function Q:

7 $$Q=\mathop{\sum }\limits_{i=1}^{n}\mathop{\sum }\limits_{j=1}^{m}{\left[\frac{{{\rm{x}}}_{ij}-\mathop{\sum }\limits_{k=1}^{p}{G}_{ik}{F}_{ki}}{{u}_{ij}}\right]}^{2}$$

where u_ij_ represents the uncertainty of HM j from sample i, and it can be calculated using Eqs. (8)-(9):

8 $${\rm{if}}\,{\rm{c}}\le {\rm{MDL}},\,{u}_{ij}=5/6\times MDL$$

9 $${\rm{or}}\,{\rm{else}},\,{u}_{ij}=\sqrt{{(errorfraction\times c)}^{2}+MD{L}^{2}}$$

### Date analysis

Descriptive statistics, such as median, mean, maximum, minimum, coefficient of variation and standard deviation, were used to characterize the contents of HMs in samples. Prior to calculation of GBCs, the K-S test was applied to determine whether HM content followed a normal distribution, and the logarithmic transformation was performed to normalize. In this work, all statistical analysis was carried out using SPSS 20 and Microsoft Excel 2007. ArcGIS 10.0 was performed to achieve the spatial maps, and the method of ordinary kriging was selected to interpolate the contents for these soil HMs.

## Results and discussion

### Content levels of soil HMs

Fundamental statistical characteristics of ten HMs in topsoils were presented in Table 1. The average contents of Cr, Hg, As, Pb, Ni, Cd, Cu, Zn, Co and V were 30.79, 0.16, 13.00, 53.89, 10.08, 0.26, 14.73, 80.38, 5.64 and 54.84 mg/kg, respectively. The average concentrations of all 10 HMs were below their corresponding national soil quality guideline values^35^. Among the samples, concentrations of Cd in 54 samples (25.47%) and Hg in 37 samples (17.25%) exceeded the Grade II values. Meanwhile, the highest contents of Cd and Hg were 4.63 and 3.50 times the corresponding secondary threshold levels, respectively. These indicated that contamination of Cd and Hg in Jiedong soil was relatively severe. However, the concentrations of other HMs were generally lower than secondary threshold levels. As for the coefficients of variation, As, Hg, Cd and Cu all have relatively high coefficients of variation, particularly As and Hg exceed 100%. These suggested that these HMs were highly likely to be affected by human activities.

**Table 1:** Statistical summary of HMs in soils of Jiedong (mg/kg).

	Cr	Hg	As	Pb	Ni	Cd	Cu	Zn	Co	V
Minimum	4.80	0.03	0.80	18.60	2.70	0.05	2.90	36.50	1.67	20.70
Maximum	117.20	1.05	120.00	122.70	34.80	1.39	63.50	316.30	16.10	131.38
Mean	30.79	0.16	13.00	53.89	10.08	0.26	14.73	80.38	5.64	54.84
Median	28.65	0.08	9.07	53.65	8.70	0.23	13.25	80.8	5.12	53.08
Standard deviation	15.86	0.16	28.71	19.17	4.47	0.16	8.75	37.65	2.75	18.30
Coefficient of variation	0.52	1.02	2.21	0.36	0.47	0.61	0.59	0.47	0.49	0.33
Grade II^a^	150	0.3	40	250	40	0.3	50	200	—	—

^a^Data from CEPA (1995).

### Establishment of GBCs

**Figure 2:**
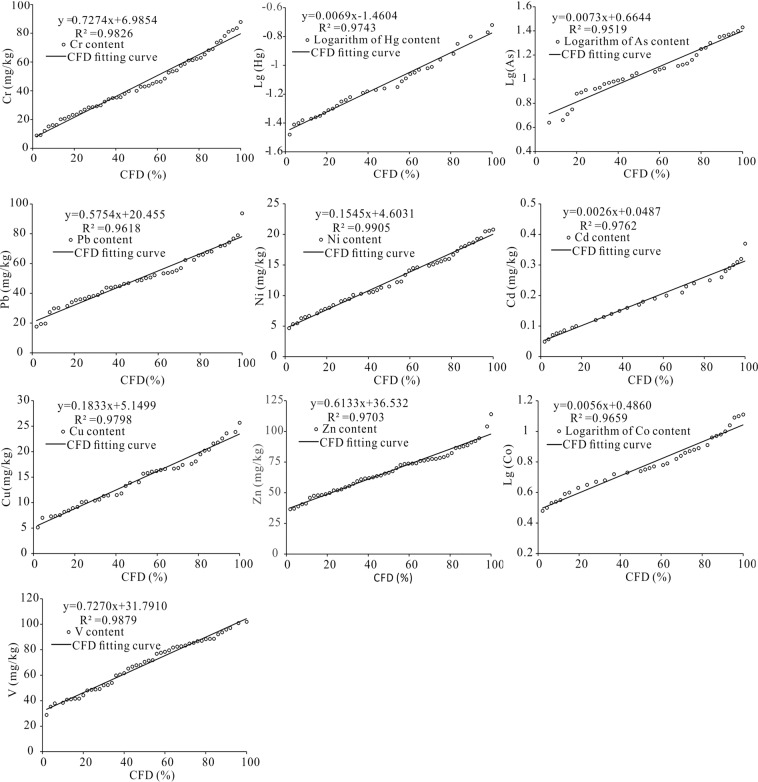
Cumulative frequency distribution curves of 10 HMs.

After passing the K-S test, the results showed that contents of HMs in subsoil samples were either normally distributed (for Cr, Pb, Ni, Cd, Cu, Zn, and V) or log-normal distribution (for Hg, As and Co). Therefore, the log-transformation was performed for the contents of Hg, As, and Co, respectively. The baseline contents of all HMs were estimated by applying CFD curves and presented in Fig. 2. The determined GBCs for Cr, Hg, As, Pb, Ni, Cd, Cu, Zn, Co and V were 39.91, 0.072, 11.48, 47.62, 12.70, 0.17, 14.22, 64.54, 6.31, and 68.14 mg/kg, respectively. The estimated baseline contents were compared with background values (BVs) of World^36^, China and Guangdong Province^37^, as well as another baseline concentration in Guangdong^38^ (Table 2). Except for Hg, As and Pb, the estimated baseline contents for Cr, Ni, Cd, Cu, Zn, Co and V in Jiedong were much less than the world soils. It was found that the determined baseline contents of Cr, Ni, Cu and Co were less than their BVs in China and Guangdong Province. Meanwhile, the estimated baseline values of As, Pb and Cd were higher than the BVs in China and Guangdong Province. For instance, the estimated baseline value of Cd was 1.75 times the BV of China and 3.04 times the BV of Guangdong Province. However, the baseline concentrations in this study were quite different from those of Guangdong Province in the study of Zhang *et al*.^38^. For example, the predicated baseline values of Pb, Cd and Zn in this study were obviously greater than those in the study of Zhang *et al*.^38^. Therefore, it is necessary to establish GBCs based on Jiedong District to evaluate local soil HM pollution.

**Table 2:** Geochemical baseline concentrations determined in Jiedong and background values in other literatures (mg/kg).

	Cr	Hg	As	Pb	Ni	Cd	Cu	Zn	Co	V
GBCs (this study)	39.91	0.072	11.48	47.62	12.70	0.17	14.22	64.54	6.31	68.14
BVs of Guangdong^a^	50.5	0.078	8.9	36.0	14.4	0.056	17.0	47.3	7.0	65.3
BCs of Guangdong^b^	57.8	0.10	—	37.5	18.0	0.10	18.0	51.4	—	—
BVs of China^a^	61.0	0.065	11.2	26.0	26.9	0.097	22.6	74.2	12.7	82.4
BVs of World^c^	59.5	0.07	6.83	27.0	29.0	0.41	38.9	70.0	11.3	129.0

^a^data from CNEMC (1990).

^b^data from Zhang *et al*. (2008).

^c^data from KabataPendias (2011).

Compared with these GBCs, the concentrations of Hg, As, Pb, Cd, and Zn in the topsoils in Jiedong District accumulated by 122%, 13%, 13%, 53%, and 25%, respectively. However, the mean content of Cu was only slightly higher than its GBC, and the average concentrations of Cr, Ni and V were lower than their corresponding GBCs.

### Evaluation of contamination and ecological risk of HMs

The results of EFs were presented in Table 3. The EF ranges of Cr, Hg, As, Pb, Ni, Cd, Cu, Zn, Co and V were 0.26–2.95, 0.32–12.31, 0.10–16.67, 0.22–6.96, 0.42–2.24, 0.36–12.12, 0.30–5.54, 0.47–12.21, 0.35–3.13 and 0.80–1.20, respectively, with an average value of 0.91, 2.54, 1.32, 1.56, 0.95, 2.01, 1.25, 1.63, 0.98 and 0.95, respectively. It was found that the mean EFs of As, Pb, Cu and Zn were in the range of 1~2, and the average EFs of Hg and Cd were greater than 2, indicating that these 6 HMs were likely to be primarily affected by anthropogenic activities. The average EFs of Cr, Ni, Co and V were <1, showing no accumulation and also revealing a natural source. The EFs for Hg in 75.94%, As in 49.53%, Pb in 77.83%, Cd in 89.15%, Cu in 63.68% and Zn in 80.66% of soil samples were >1, suggesting that these HMs widely enriched in Jiedong. Especially for Hg and Cd, Hg in 13.68% and Cd in 2.36% of all samples were major enrichment. In addition, the highest EF values of As (16.67) and Zn (12.21) showed major enrichment. All of these indicated that the soil HMs in Jiedong District were mainly slightly to moderately enriched, which also meant soils in Jiedong District were generally influenced by human activities.

**Table 3:** EF, CF and E_i_ values of HMs in soils of the study area.

		Cr	Hg	As	Pb	Ni	Cd	Cu	Zn	Co	V
EF	Minimum	0.26	0.32	0.10	0.22	0.42	0.36	0.30	0.47	0.35	0.80
	Maximum	2.95	12.31	16.67	6.96	2.24	12.12	5.54	12.21	3.13	1.20
	Mean	0.91	2.54	1.32	1.56	0.95	2.01	1.25	1.63	0.98	0.95
CF	Minimum	0.12	0.37	0.07	0.40	0.21	0.33	0.20	0.57	0.26	0.30
	Maximum	2.93	14.62	10.45	2.58	2.74	8.44	4.47	4.90	2.55	1.93
	Mean	0.77	2.19	1.13	1.13	0.79	1.56	1.04	1.25	0.89	0.80
E_i_	Minimum	0.24	14.73	0.69	1.95	1.06	9.86	1.02	0.57	1.32	0.61
	Maximum	5.87	584.77	104.53	12.88	13.70	253.09	22.33	4.90	12.75	3.86
	Mean	1.54	87.42	11.33	5.66	3.97	46.87	5.18	1.25	4.47	1.61

The CFs of HMs were calculated and recorded in Table 3. The CF values for the selected HMs decreased as follow: Hg > Cd > Zn > As = Pb > Cu > Co > V > Ni > Cr. The CFs of Hg and Cd were relatively high, indicating that Hg and Cd in the study area exhibited moderate contamination. The average CFs of Cr, Ni, Co and V were <1, inferring no pollution of these 4 HMs, while the average CFs of As, Pb, Cu and Zn suggested slight pollution. The PLI value, based on further calculation of the CFs of 10 HMs, was useful to overall evaluate the level of soil HM pollution in Jiedong. The PLI values ranged from 0.47 to 2.22. There were 123 sampling sites with PLI values lower than 1, 87 sampling sites with PLI values ranging from 1 to 2, and 2 sampling sites with PLI values ranging from 2 to 5. The above revealed that approximately 58.02% of the sampling sites were not polluted by HMs, 41.04% of the sampling sites were moderately polluted, and 0.94% of the sampling sites were severely polluted.

Considering that different pollutants have different toxicity to human body, RI was used to comprehensively evaluate the ecological risks of soil HMs. Single risk indexes (E_i_) were shown in Table 3. The mean E_i_ values of ten HMs were ranked according to intensity of ecological risk as: Hg » Cd » As > Pb > Cu > Co > Ni > V > Cr > Zn. Based on the classification criteria of Hakanson^33^, the mean E_i_ values of Hg (87.42) and Cd (46.87) belong to considerable and moderate single potential ecological risk, respectively. Nevertheless, the mean E_i_ values for Cr, As, Pb, Ni, Cu, Zn, Co and V were <40, indicating these 8 HMs show the low degree of potential ecological risk. Integrated potential ecological risk assessment of 10 HMs presented that RI values in 58.96% of the sampling sites were <150, suggesting that the ecological risks in most areas of the region were low. The RI values of 61 sampling sites ranged from 150 to 300, inferring that 28.77% of the study area had medium ecological risk. And the RI values of 24 sampling sites were in the range of 300 and 600, indicating a significant ecological risk in 11.32% of the study area. However, it is worth noting that 0.94% of the area faced an extreme ecological risk from soil HMs.

### Spatial distribution of HMs in soils

**Figure 3:**
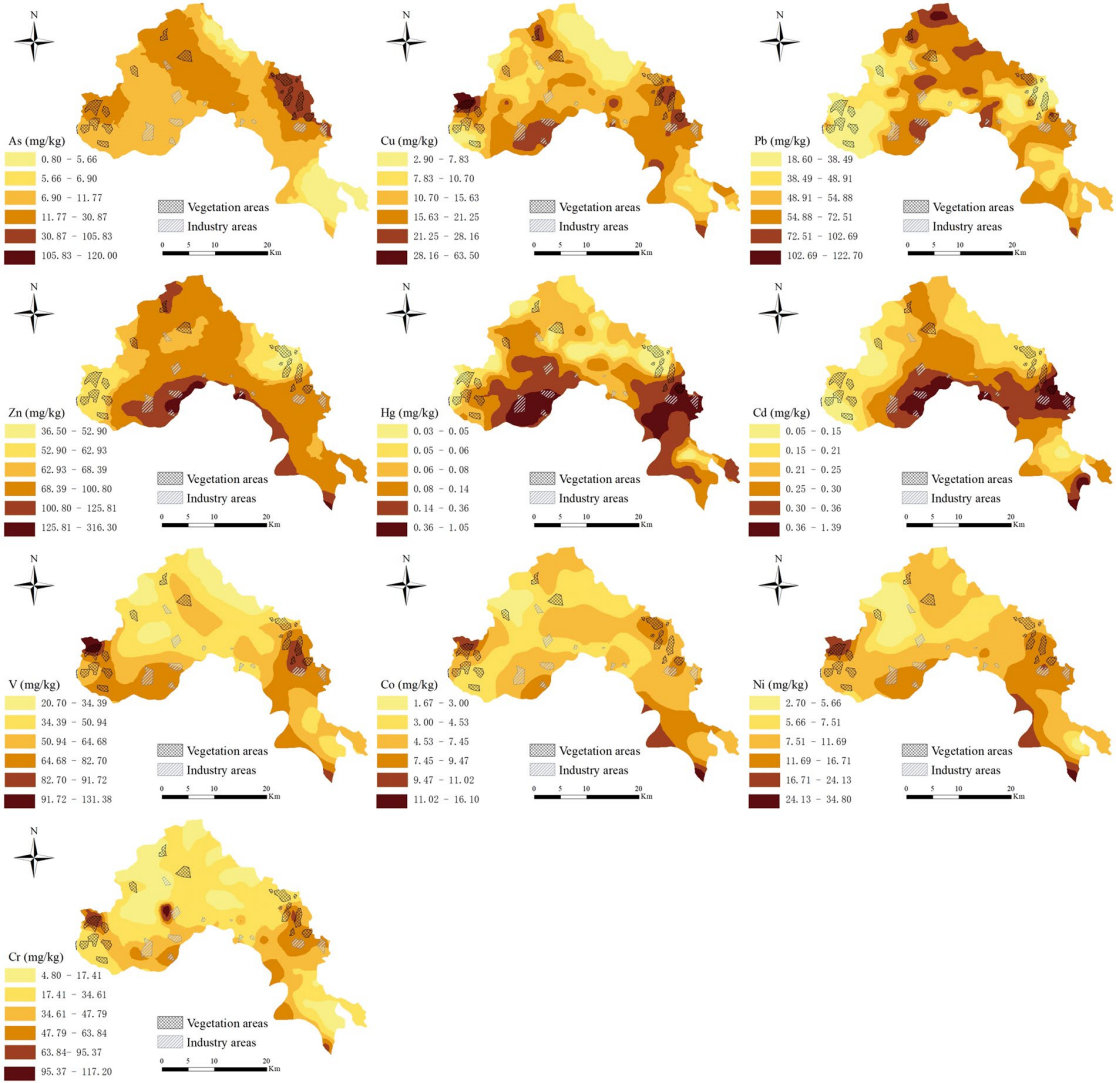
Spatial distribution of 10 HMs in soils across study area.

The spatial distributions of HM content are useful to distinguish hotspots and identify the possible sources of HMs in soils. The spatial variations of 10 HMs in Jiedong District were presented in Fig. 3. Spatial variation tendencies of Cu and As were approximately similar, and these hot spots were situated at the western and eastern parts of the study area, where agricultural production regions were concentrated. inputs from agricultural activities, for instance, fertilizers, herbicides and pesticides, contain large amounts of As and Cu^39,40^, which contribute to the accumulation of As in the soil of Jiedong District.

As presented in Fig. 3, spatial variations of Pb and Zn were highly consistent, and high content areas of Pb and Zn were found at the middle of Jiedong District, which suggested that Pb and Zn may come from the same origin. These hotspots of Pb and Zn were observed near major roads and urban areas where traffic and population were quite intensive. It is reported that common origins of Pb and Zn are traffic emissions^41,42^. In addition, Zn as a necessary micronutrient for plant, excessive or insufficient zinc in soil will affect plant growth. According to the spatial distribution of Zn, the eastern crop growing area of Jiedong District may face Zn deficiency, while the southern area may face Zn excess.

The spatial trends of Hg and Cd contents were highly similar and relatively concentrated (Fig. 3). Soils with high concentrations of Hg and Cd were detected in the south and east of Jiedong District. Major industrial parks, such as textile mills, pharmaceutical factories and metal manufacturing factories were widely distributed in these areas. These related industries have been demonstrated to cause the enrichment of Hg and Cd in soils via atmospheric deposition^43,44^.

According to Fig. 3, it revealed the overall downward trend of V, Co, Ni and Cr concentrations from southern to northern. The high values of these 4 HMs were focused on the southwest and southeast edge of Jiedong District, where most of them were natural vegetation areas and were less influenced by anthropogenic activities. Additionally, the average concentrations of V, Co, Ni and Cr were less than their GBCs, and their EF values were also low. Therefore, it suggested that V, Co, Ni and Cr may have the same origin, which was determined by the parent material.

### Source apportionment by PMF

**Figure 4:**
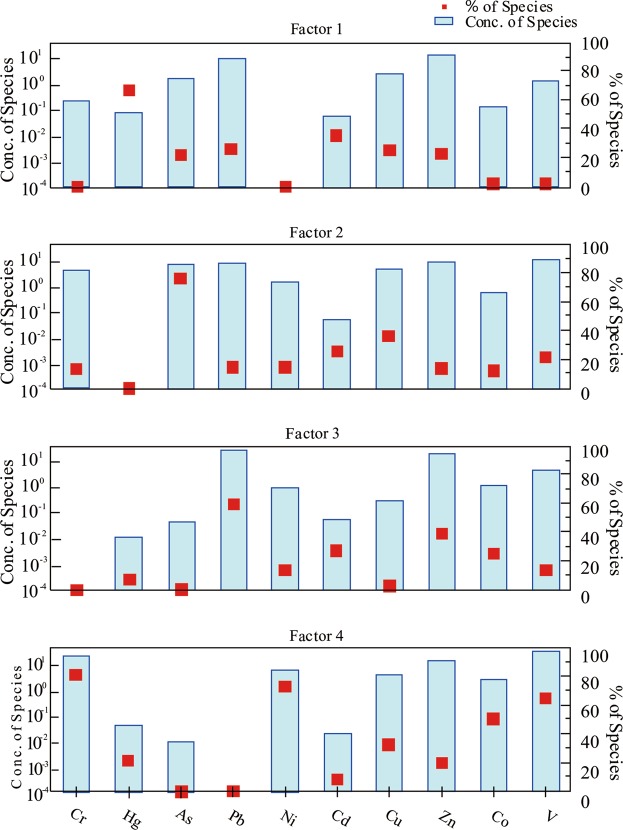
Factor profiles and contribution percentage of HMs from PMF model.

The input files of PMF model contained concentration data of 10 HMs in 212 samples and uncertainty data associated with these concentrations. The most appropriate number of factor solutions were chosen according to the minimum and steady Q value, and the optimal factor number, that is, four factors were achieved. Here, the residual value of most soil samples was in the range of −3~3. The R^2^ values represented determination coefficients of 10 HMs, all of which were greater than 0.75. These indicated that the results were reliable, and four factors produced by PMF operation were presented in Fig. 4.

Factor 1 was dominated by Hg (64.76%) and Cd (35.07%) (Fig. 4), which accounted for 20.61% of the total variance. Elements Hg and Cd had relatively high coefficients of variation, indicating that they were greatly influenced by human activities. Meanwhile, Hg in 75.94% of the samples and Cd in 89.15% of the samples were accumulated, and the highest EF exceeded 10, which belonged to major accumulation. All these inferred that Hg and Cd might come from anthropogenic sources. Dvonch *et al*.^45^ reported that about 80% of total Hg originated from human activities, and the vast majority of Hg was released into the atmosphere in the form of vapor. Meanwhile, previous studies have presented that Hg enrichment in soils is mainly related to industrial activities, such as the burning of fossil fuels, smelting, and the manufacture of chemical raw materials and chemicals^25,46,47^. Cadmium is mainly used in the manufacture of batteries, pigments, alloys and electroplating, and as a stabilizer for plastic products. It is therefore generally regarded as the marker element of industry^48^. According to the survey of JPGP (Jieyang People’s Government Portal)^49^, textile and garment industry, rubber and plastics manufacturing, metal smelting and manufacturing accounted for 8.24%, 14.56% and 17.88% of the total industry in Jiedong District, respectively. Moreover, there were a number of chemical plants and pharmaceutical factories in the study area. Therefore, the waste water, waste gas and waste residue generated by all these industries were the major origins of soil Hg and Cd in Jiedong. As analyzed above, Factor 1 could be determined as industrial activities.

Factor 2 accounted for 24.20% of the source contribution, and was predominated by As (77.90%) and Cu (37.83%) (Fig. 4). As mentioned above, the average EFs of As and Cu were >1, and the maximum EFs of As and Cu were 16.67 and 5.54, respectively, both of which were major enrichment. Moreover, the spatial distributions of As and Cu revealed that their high-value regions were distributed in the eastern part of Jiedong District, which was consistent with the agricultural planting areas. Arsenic and Cu are often associated with agricultural practices, including the use of pesticides and fertilizers. Pesticides or herbicides used in agricultural production contain large amounts of inorganic arsenic compounds, such as sodium arsenate and calcium arsenate^18^. Many researches^50,51^ have shown that As was predominantly found in phosphate fertilizers, which is also an important pathway for As to seep into the soil. Livestock manures and pesticides are the major origins of Cu in soils. Copper and its compounds are mainly applied in fungicides and pesticides^23^. It is investigated that 69% of Cu in soils is derived from livestock manure^52^. According to the statistical yearbook^49^, the application of pesticides and fertilizers applied to cultivated land in Jiedong District was 80 kg/ha and 3140 kg/ha, respectively. Furthermore, in China, the utilization rate of pesticides and fertilizers is commonly low, about 70% of which is lost to the surroundings^23^. Consequently, Factor 2 was defined as agricultural practices.

For the third factor, it was identified by Pb (58.40%) and Zn (42.08%) (Fig. 4), which explained 19.22% of the total contribution. The concentrations of Pb and Zn in most soils were greater than GBCs, especially for Zn, which was 1.53 times the GBC. Meanwhile, the EFs of Pb and Zn showed a relatively high level of enrichment. These indicated that Pb and Zn in Jiedong District came from human input. Normally, Pb is considered as a sign of transportation^46^. Although leaded gasoline has been banned since 2000, Pb enrichment still exists due to the use of leaded gasoline for several decades^53^. While braking device and clutch wear of automobile are other origins of Pb^54^. As for Zn, the abrasion of vehicle tyres and the corrosion of galvanized of vehicle components lead to the enrichment of Zn in the soil^41,55^. Xia *et al*.^56^ reported the contents of Pb and Zn in roadside soil were obviously positively correlated with transportation density. Besides, according to the spatial distribution of Pb and Zn, the contents of Pb and Zn were high near those main roads (expressways and national highways) (Fig. 3). Similar results have been summarized in many other researches^2,10,57^. From what has been discussed above, Factor 3 was considered as traffic emissions.

Factor 4 was characterized by descending order of Cr, Ni, V, Co and Cu, with contribution rates of 81.03%, 71.60%, 63.69%, 53.87% and 31.86%, respectively (Fig. 4). This source was the biggest factor and taken over 35.97% of the total variance. Except that the mean content of Cu was slightly higher than its GBC, the averages of the other 4 HMs were lower than their corresponding GBCs. Moreover, the EF values of these HMs revealed that most soils had no accumulation. For example, the ratio of EF values < 1 in the study area presented 66.98% of Cr, 66.68% of Ni and 68.40% of V. All these indicated that this group of HMs came from a natural origin. Previous researches have similar conclusions. Franco-Uría *et al*.^58^ studied soil HMs in Pastoriza (Spain), and found that Cu, Ni and Co were correlated with two lithogenic components. Cai *et al*.^59^ reported that Co, Cr, and Ni were related to soil parent material. In addition, the study from the Lv *et al*.^25^ in Ju Country (China) suggested V, Ni, Cr, Co and partially Cu originated from natural source. Thus, Factor 4 was classified as a natural source.

Fig. S1 illustrated the percentage of four sources in the total variance percentage. Natural source (35.97%) was assigned as the biggest contribution rate of HMs in the soil of Jiedong District, followed by agricultural practices (24.20%), industrial activities (20.61%) and traffic emissions (19.22%). In short, anthropogenic sources (64.03%) dominated the total contribution rate, which was the main factor affecting the content of soil HMs in Jiedong District. The use of pesticides, livestock manures and fertilizers, textile and garment processing, plastic and rubber production, metal manufacturing, and vehicle emissions were considered as the major origins of HM contamination in Jiedong District. In other words, anthropogenic activities posed a tremendous influence on the accumulation of soil HMs in Jiedong District. Therefore, in order to control and alleviate the contamination of soil HMs in Jiedong, agricultural production, industrial activities, and transportation should be regulated and adjusted.

## Conclusion

In this research, the GBCs of Cr, Hg, As, Pb, Ni, Cd, Cu, Zn, Co and V were established, which were 39.91, 0.072, 11.48, 47.62, 12.70, 0.17, 14.22, 64.54, 6.31, and 68.14 mg/kg, respectively. The average concentrations of Hg, As, Pb, Cd, Cu and Zn were greater than corresponding GBCs, especially for Cd and Hg, which were 1.53 and 2.22 times their GBCs. Meanwhile, the concentrations of Hg in 37 samples (17.25%) and Cd in 54 samples (25.47%) exceeded the Grade II values. The EF and PLI suggested moderate HM pollution, but contamination of Hg and Cd was more serious. And the RI values showed that the potential ecological risks were low in most parts of the total area. Analysis based on EF, geostatistics and PMF, four sources were identified. Lead and Zn were mainly related to traffic emissions. Mercury and Cd mainly derived from industrial activities, such as textile and garment processing, plastic and rubber production and metal manufacturing. Arsenic and part of Cu mainly came from agricultural inputs, including the use of fertilizers, livestock manures and pesticides. Chromium, Ni, V, Co, and part of Cu were originated from natural source associated with parent materials. This research highlighted the demand to determine regional GBCs as an important foundation for accurate HM pollution evaluations. Furthermore, this study also offers a reference for preventing and controlling soil HM pollution by proposing the management of these anthropogenic sources.

## Supplementary information

Supplementary information

## Data Availability

The data in this article is available.
