# Supplementary material for: Characterizing pollution and source identification of heavy metals in soils using geochemical baseline and PMF approach
Source: Sci Rep. 2020 Apr 15;10:6460. doi: 10.1038/s41598-020-63604-5 (PMC7160138; doi:10.1038/s41598-020-63604-5)
Supplement: Supplementary file 1 — Supplementary information [file 41598_2020_63604_MOESM1_ESM.doc]

***Supplementary Information***

**Characterizing pollution and source identification of heavy metals in soils using geochemical baseline and PMF approach**

Hui-Hao Jiang ^a,b^, Li-Mei Cai a,b,c[[1]](#footnote-2)*, Han-Hui Wen ^d^, Jie Luo ^a,b^

^a^ *Key Laboratory of Exploration Technologies for Oil and Gas Resources (Yangtze University), Ministry of Education, Wuhan 430100, China*

^b^ *College of Resources and Environment, Yangtze University, Wuhan 430100, China*

*^c^ Key Laboratory of Mineralogy and Metallogeny, Guangzhou Institute of Geochemistry, Chinese Academy of Sciences, Guangzhou 510640, China*

*^d^ No.940 Branch of Geology Bureau for Nonferrous Metals of Guangdong Province, Qingyuan 511500, China*

Fig. S1. Percentage contributions of four source factors from the PMF model.

1. * Corresponding author.

   E-mail addresses: clmktz88@yangtzeu.edu.cn (L.M. Cai). [↑](#footnote-ref-2)
